# Renal failure following insulin purging in atypical anorexia nervosa and type 1 diabetes mellitus

**DOI:** 10.3389/fpsyt.2023.1325021

**Published:** 2023-12-11

**Authors:** Caroline Rometsch, Martina Guthoff, Stephan Zipfel, Andreas Stengel

**Affiliations:** ^1^Department of Experimental and Clinical Medicine, University of Florence, Florence, Italy; ^2^Department of Psychosomatic Medicine and Psychotherapy, University Hospital Tübingen, Tübingen, Germany; ^3^Department of Internal Medicine IV, Diabetology, Endocrinology, Nephrology, University Hospital Tübingen, Tübingen, Germany; ^4^German Center for Mental Health (DZPG), Tübingen, Germany; ^5^Charité Center for Internal Medicine and Dermatology, Department for Psychosomatic Medicine, Charité - Universitätsmedizin Berlin, Corporate Member of Freie Universität Berlin, Humboldt-Universität zu Berlin and Berlin Institute of Health, Berlin, Germany; ^6^Freie Universität Berlin, Humboldt-Universität zu Berlin and Berlin Institute of Health, Berlin, Germany

**Keywords:** anorexia nervosa, insulin purging, diabetes mellitus, psychosomatic medicine, renal failure, atypical anorexia nervosa, nephrology

## Abstract

**Objective:**

Anorexia nervosa (AN) and atypical anorexia nervosa (AAN) are severe and complex eating disorders that can be prevalent among individuals with type 1 diabetes mellitus (T1DM). Insulin purging, characterized by the intentional underuse / omission of insulin to control weight, is under-recognized in medicine and is a purging strategy of patients with AN or AAN and comorbid T1DM. Often, this can lead to renal failure, necessitating a (pancreas-) kidney transplantation. This article presents a comprehensive overview of the interplay between AN/AAN and T1DM and summarizes the evidence in literature.

**Methods:**

A narrative review is presented on basis of a detailed case study of a 32-year-old female with end-stage renal failure seeking (pancreas-) kidney transplantation displaying etiology, diagnosis, comorbidities, complications, and treatment of AN and AAN with emphasis on those patients with T1DM.

**Results:**

Insulin purging in patients with AN/AAN and coexisting T1DM can exacerbate T1DM complications, including accelerating the onset of end-stage renal failure. A multidisciplinary approach including nutrition treatment and psychotherapeutic techniques was considered necessary for treatment, focusing on psychosomatic in-patient care before and after organ transplantation.

**Conclusion:**

Insulin purging in patients with AAN and T1DM poses severe health risks, including accelerated renal complications. For those considering transplantation, insulin purging has explicitly to be diagnosed and a holistic treatment addressing both the renal condition and psychosomatic symptoms/disorders is crucial for successful post-transplant outcomes.

## Clinical case

**Figure 1:**
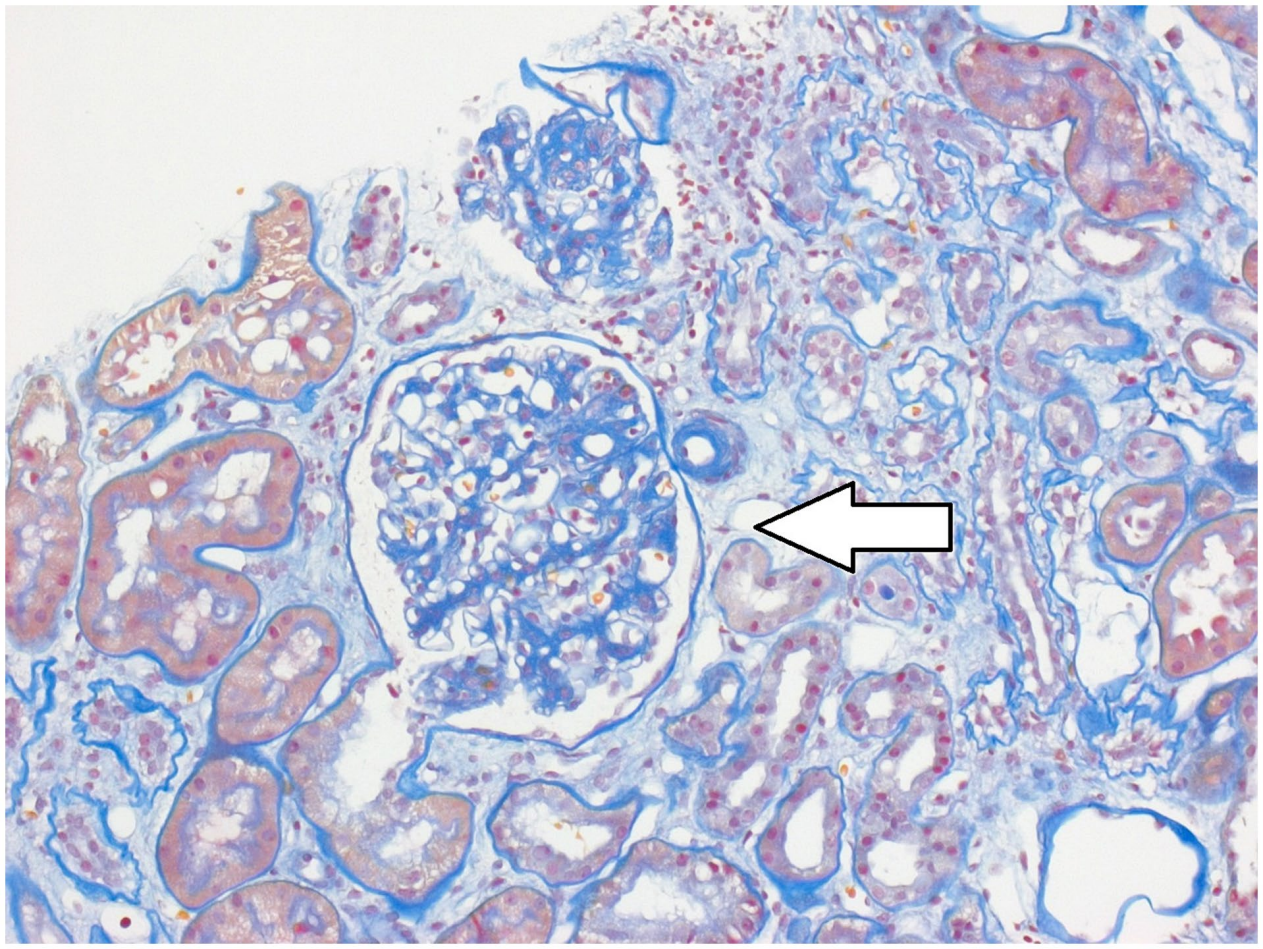
Representative image of the kidney from the case presented. Hematoxylin and Eosin stain, arrow indicates diabetic nephropathy.

A 32-year-old female patient was presented for a transplant assessment due to end-stage renal failure. She was on life-sustaining hemodialysis, adhering to the standard regimen of three sessions per week. However, the time commitment posed limitations for her. She sought to work full-time after completing her studies, which led her to seek a (pancreas-) kidney transplant evaluation at the University Hospital of Tübingen, Germany. Her medical history showed that she had type 1 diabetes mellitus (T1DM), since early childhood. Further histological tests showed evidence of diabetic nephropathy (see Figure 1). The transplant assessment included both an in-depth nephrological evaluation including imaging and a psychosomatic assessment to detect any potential mental health issues to ensure treatment adherence after transplantation as recommended in the current guideline (1).

During the psychosomatic assessment, a low body weight with an actual BMI of 18.8 kg/m^2^ was reported by the female patient. She stated ritualistic eating behaviors with food avoidance and preferences of low-calorie food. She cooked diverse high-calories dishes, spending a huge amount of time for preparing, finally not consuming it. She exhibited an intense fear of gaining weight, and her perception was skewed, believing she was overweight. In past, she reported self-induced vomiting and the misuse of laxatives to control her weight. She did suffer from amenorrhea. During her assessment, the female patient exhibited clear signs of depression. She seemed sad, expressing feelings of worthlessness and hopelessness. She lost interest in activities she once enjoyed, and she lacked from motivation. Moreover, she struggled with sleep and had difficulty concentrating and making decisions.

As documented in the patient’s file, a severe case of anorexia nervosa (AN) and a comorbid depression was described, with multiple treatments beginning from her 14^th^ year. During the patient’s medical history assessment, it was disclosed that she had not adhered to the prescribed diabetic treatment regimen, particularly concerning the administration of insulin. Her deliberate practice involved self-administering minimal or no insulin, a strategy known as “insulin purging,” with the primary objective of inducing glucosuria as a means to manage her weight. The poor blood sugar control likely contributed to a faster progression of the diabetic nephropathy. As a result of the psychosomatic evaluation, the patient was diagnosed with an atypical anorexia nervosa (AAN) and a moderate depression. Insulin purging represents a potential complication that can manifest in patients diagnosed with AAN, as well as in individuals presenting with the coexistence of AN and T1DM. During the multidisciplinary transplant conference, psychosomatic treatment and follow-up care were deemed essential and seen as prerequisites for a (pancreas-) kidney transplant. The patient agreed on attending the treatments to finally be listed for organ transplantation*.

*The case report was composed to ensure that identifiable information was excluded.

## Insulin purging and its associated complications

“Diabulimia” and “insulin purging” are terms used to describe the coexistence of AN or AAN with diabetes mellitus (2). Individuals with this condition intentionally underuse / omit insulin to manipulate weight. By not administering the required insulin, patients induce hyperglycemia (measurable by an elevated HbA1c) and glucosuria, which can result in weight loss over time. Physiologically, insufficient insulin disrupts carbohydrate metabolism and fat formation, spurring tissue breakdown for ketone production. This, combined with osmotic diuresis, causes dehydration and contributes to further weight loss. On the other side, due to food restrictions, the clinical risks of hypoglycemia exists (2). Insulin purging frequently manifests as a complication in individuals who have both AN/AAN and comorbid T1DM. There is limited research available that addresses the prevalence of insulin misuse for weight regulation when AN or AAN co-occur with type T1DM, with estimates suggesting it occurs in approximately 40–50% of such cases (3, 4). To the best of our knowledge, there is no epidemiological study on the prevalence.

Diabetic complications like nephropathy, neuropathy, and retinopathy have been linked to pathological eating behaviors showing that these behaviors nearly triple the risk of such complications (5) and of diabetic ketoacidosis (6). Referring to the presented case report, insulin restriction in diabetes mellitus results in higher rates of complications and an overall increased risk of mortality (7) when comorbid AN was diagnosed (8).

## Anorexia nervosa and atypical anorexia nervosa – prevalence, etiology, and diagnostic criteria

AN is a severe psychiatric disorder that can impact individuals regardless of age, gender, or ethnicity (9). The point prevalence of AN was estimated with 2.8% for females (range of 0 to 4.8%) and 0.3% for males (range of 0 to 0.4%) (10). Life time prevalence is reported with rates up to 4% in females and 0.3% in males (11). The incidence of AN varies between 0.5 and 318.9 cases per 100,000 women-years. This considerable variability can be attributed to the diverse diagnostic criteria and assessment tools employed across different studies and healthcare settings (12). Although the rate has remained stable over time, there has been an increase in the incidence of AN among individuals aged 15 years and younger (11). The etiology of AN encompasses genetic factors (rates between 28 and 74% (13)), neurobiological influences, developmental elements, and environmental factors (9). The common custom taxonomies - Diagnostic and Statistical Manual of Mental Disorders (DSM) and International Classification of Diseases (ICD) - define AN by referencing low body weight, an intense fear of weight gain, and a disturbance in the perception of body weight. Notably, endocrine imbalance/amenorrhea was included as a criterion in the DSM-IV and ICD-10; however, while no longer being a criterion in the DSM-5.

In contrast, AAN was defined historically to account for not eating due to psychiatric reasons, such as a fear of poisoning or ascetic denial (14, 15). Nowadays, AAN is considered a subtype of eating disorder and applied according to the DSM-5 under the category of Other Specified Feeding or Eating Disorders (OSFED). Diagnostic criteria of AAN are in line with those for AN (restricting behaviors, over-exercising, binging or purging, fear of being overweight, and weight loss) with a key distinction being the body weight. Despite significant weight loss and restrictive behaviors, individuals with AAN are not necessarily below the expected body weight for their age, sex and developmental trajectory (16). Notedly, patients might still be malnourished due to their restrictive behaviors and significant weight loss (16). AAN occurs with higher point prevalence rates compared to AN ranging between 0.2 and 13.0%, and lifetime prevalence rates between 0.2 and 4.9% (6).

Even though the current patient was diagnosed with AAN following DSM criteria, it is crucial to acknowledge ongoing debates in the field. Recently, the appropriateness of applying the AAN diagnosis to patients in partial remission from AN has been questioned. Current discussions suggest excluding individuals with a lifetime diagnosis of AN to refine the diagnostic criteria, thereby improving clinical utility and treatment outcomes (17).

## Psychopathology, complications, and comorbidities of AN and AAN

Psychopathological features specific to AN and AAN, such as restraint, eating habits, concerns about shape and weight, drive for thinness, and body dissatisfaction, are more pronounced in AAN than in AN (18). On the other hand, non-disorder-specific psychopathological aspects, including functional impairment/quality of life, self-esteem, weight-related obsessive-compulsive behaviors, suicidality, impulsivity, dysfunctional metacognitions, and personality styles do not show a distinction between AN and AAN (18).

Medical complications of AN are numerous and might occur as rare extreme organ dysfunction and atypical signs (19). Most important complications are: cardiac complications (e.g., arrythmia, orthostatic hypotension, myocardial atrophy), electrolyte imbalance (e.g., hyponatremia, hypokalemia), endocrine imbalance (e.g., hypogonadotropic hypogonadism, osteopenia), gastroenterological (gastric dysmotility, delayed gastric emptying), integumentary (e.g., lanugo, hair loss, calluses from purging, hypercarotenemia) as well as dental complications, and not last the important, highly mortal refeeding syndrome, which is caused by hypophosphatemia (causing seizure, delirium) (16). In patients with a long lasting AN, renal complications are frequent and can lead to urinary urgency, nocturnal enuresis, hypokalemia, hyponatremia, hypomagnesemia, hypophosphatemia, urinary lithiasis and ultimately renal failure (20).

AN and AAN were found to have similar prevalence rates regarding the variables history of trauma, experiences of childhood abuse (whether sexual, physical, or emotional), the number of psychiatric diagnoses, and the occurrence of suicidal thoughts/attempts (21). Further on, meta-analyses indicated that levels of depression and anxiety were similar between AN and AAN (18, 21). The relationship between depression and eating disorders has been extensively studied. It has been determined that depression can be a risk factor for eating disorders and conversely, eating disorders can predispose individuals to depression (22). Depression was hereby identified to be a negative prognostic factor for weight gain (23).

The prevalence of AN in patients with T1DM was also meta-analyzed resulting in 0.3% which was not significantly different from individuals without AN (24). However, the last few years have seen a surge in the prevalence of the condition in patients with T1DM, with some estimates suggesting a rate as high as 7%. Contributing to this trend could be a perceived loss of autonomy over the eating behavior, in addition to the psychological impacts of anxiety and depression, which are thought to play a role in the rising prevalence (2).

## Treatment options for AN/AAN and T1DM

To date, there are no specific guidelines addressing the co-management of AN/AAN and T1DM. However, the current case illustrates the necessity for an interdisciplinary approach, bringing together expertise from endocrinology, nephrology, internal medicine, and other relevant specialties, in conjunction with psychosomatic medicine or, when unavailable, psychiatry or clinical psychology to address diabetes mellitus, AN/AAN, and comorbid affective disorders.

The fundamental components for AN treatment, namely nutritional intervention and psychotherapeutic support (9), must be accurately adjusted when addressing the specific challenges in patients with comorbid T1DM (16). Ensuring a consistent and adequate caloric intake (16), particularly in medically restricted diets, helps in curbing binge eating tendencies (2). Regular monitoring of blood glucose levels is essential, and patients are advised to adjust insulin doses in relation to carbohydrate intake (2). Advanced diabetic technologies, like continuous subcutaneous insulin infusion, present a promising option for improved blood sugar regulation (23, 25).

While established psychotherapeutic strategies for AN/AAN are recognized, there is an apparent gap in psychotherapeutic guidance specifically tailored to the comorbid presentation of AN/AAN and T1DM. Drawing from the existing body of research on AN/AAN, such knowledge could serve as a preliminary foundation for future research and the formulation of more comprehensive treatment guidelines. Current literature on AN/AN cites various therapeutic modalities including enhanced cognitive behavioral therapy, focal psychodynamic psychotherapy, the Maudsley model for adults, and specialist supportive clinical management. These therapies have shown moderate efficacy in facilitating weight gain and alleviating psychopathological symptoms (9, 16). Recent findings indicate that behavioral family system therapy effectively augmented body weight, and conjoint family therapy demonstrated superior efficacy in mitigating depressive symptoms. Investigations utilizing both family and individual therapeutic modalities, especially those with extended treatment periods, produced larger effect sizes (26). Inpatient care, integrating cognitive behavioral therapy, psychoeducation, and family therapy, has been identified as the most effective treatment strategy (27).

In cases of end-stage renal disease, renal replacement therapy is indicated in approximately 5–10% of cases. Clinically diagnosed acute kidney injury (AKI) is characterized by a sudden decline in kidney function. It is crucial to emphasize that AKI is not solely defined by oliguria or anuria; instead, diagnostic criteria primarily rely on changes in serum creatinine levels and urine output (28). When facing end-stage renal disease and hemodialysis as a complication of AN/AAN and T1DM, (pancreas-) kidney transplantations might be a last option to ensure survival of the patient (29).

However, the existing literature scarcely covers solid organ transplantation in AN/AAN patients with T1DM. It is worth noting that, to date, there is a lack of treatment recommendations specifically targeting AAN coexisting with T1DM. Although AN and AAN have both distinct and overlapping features (21), the authors of this review recommended for the clinical case presented to adopt the gold standard treatment for AN. This approach should be individualized to the patient’s specific needs in regard to the renal failure, necessitating a psychosomatic in-patient multimodal treatment prior to organ transplantation, and a stable outpatient psychotherapeutic network for post-transplantation support. Regular follow-up examinations by the transplantation team after the procedure are standard practice.

## Conclusion

In patients with coexisting T1DM and AN/AAN, there exists a rare but significant risk of insulin purging leading to severe renal complications. Nonetheless, there is a gap in systematically documenting the prevalence rates of insulin purging in such cases. When renal failure is present and transplantation is being considered, it is crucial to identify behaviors tied to insulin restriction, often seen in serious psychosomatic conditions like eating disorders and affective disorders. Prior to authorizing transplantation, comprehensive treatment of these multifaceted disorders must be ensured, as well as in subsequent care. As of the present, comprehensive treatment protocols and guidelines that offer the most effective treatment procedures for patients who exhibit insulin purging while having the co-occurrence of AN/AAN and T1DM, along with end-stage renal failure, are notably absent. This gap highlights the need for further research and the development of tailored treatment approaches to address the complex needs of this specific patient population.

## Data availability statement

The original contributions presented in the study are included in the article/supplementary material, further inquiries can be directed to the corresponding author.

## Ethics statement

Ethical approval was not required for the study involving humans in accordance with the local legislation and institutional requirements. Written informed consent to participate in this study was not required from the participants or the participants’ legal guardians/next of kin in accordance with the national legislation and the institutional requirements. Written informed consent was obtained from the individual(s) for the publication of any potentially identifiable images or data included in this article.

## Author contributions

CR: Conceptualization, Data curation, Formal Analysis, Investigation, Methodology, Visualization, Writing – original draft, Writing – review & editing. MG: Writing – review & editing. SZ: Writing – review & editing. AS: Conceptualization, Methodology, Visualization, Writing – review & editing.
